# Psychiatric Disorders in Complex Regional Pain Syndrome (CRPS): The Role of the Consultation-Liaison Psychiatrist

**DOI:** 10.1155/2018/2894360

**Published:** 2018-10-17

**Authors:** Michael Brinkers, Paulina Rumpelt, Anke Lux, Moritz Kretzschmar, Giselher Pfau

**Affiliations:** ^1^Department of Anaesthesiology and Intensive Care Medicine, Otto-von-Guericke-University Magdeburg, Magdeburg, Germany; ^2^Department of Diagnostic and Interventional Radiology, Oberlausitz-Kliniken, Bautzen, Germany; ^3^Department for Biometrics and Medical Informatics, Otto-von-Guericke-University Magdeburg, Magdeburg, Germany

## Abstract

**Background:**

Complex regional pain syndrome (CRPS) is a multifactorial disorder with complex aetiology and pathogenesis. At the outpatient pain clinic of Magdeburg University Hospital, all patients, without exception, are subject to permanent psychiatric care delivered by a consultation-liaison psychiatrist. In CRPS, psychological stabilization and treatment of the neuropathic aspects are equally important. The aim of this single-center retrospective study was to determine mental/psychiatric defects impairing pain processing at the time of investigation and show the effects of treating mental disorders and neuropathic pain with the same psychotropic drugs.

**Method:**

On admission, the consultation-liaison psychiatrist examined the mental state of every patient in a semistructured interview according to AMDP (working group for methods and documentation in psychiatry). Due to the model of the Department of Anaesthesiology, we are able to compare the group of CRPS patients with all other outpatients treated for pain.

**Results:**

The medical treatment of psychiatric dysfunction leads to an analgesic effect. Only every second CRPS patient had an additional psychiatric diagnosis, and 15.6% were diagnosed with depressive mood disorders and show a higher prevalence of depressive symptoms than the general population and exceed the mean for all patients treated in our pain clinic.

**Conclusions:**

In neuropathies, treatment of the neuropathic pain has a modulating effect on mental disorders. As CRPS patients are frequently affected by depressions, and owing to the connection between depression and suicidal tendencies, patients should be seen by a consultation-liaison psychiatrist, and nonpsychiatrists should pay special attention to this patient group.

## 1. Introduction

The complex regional pain syndrome (CRPS) can be divided into two groups:

- CRPS I, formerly known as reflex sympathetic dystrophy (RSD)—here, no nerve lesions can be identified.
- CRPS II (formerly known as causalgia). The symptoms of this syndrome include evidence of a nerve lesion [1].

As CRPS belongs to the neuropathic diseases, the patients were subject to drug therapy, with special attention paid to the administration of anticonvulsant and antidepressant drugs. Wertli et al. [2] performed a meta-analysis and concluded that anticonvulsant and antidepressant drugs had no satisfying effect. By contrast, psychiatric drugs are indicated in the case of psychiatric alterations, such as agitation, anxiety, or depressive moods. CRPS in association with psychiatric disorders has only been investigated in limited studies, as the patients rarely undergo a routine and thorough psychological-psychiatric examination [3]. Rommel et al. recommend a psychological cotreatment of the patients. On the one hand, psychiatric alterations, mainly depression and anxiety, are registered in this way. On the other hand, these comorbidities should be treated with psychotherapy and/or medication. However, the question of whether psychological factors should be regarded as risk factors for CRPS is a highly controversial issue [4–6].

The care by a consultation-liaison (CL) psychiatrist is a permanent feature of the Department of Anaesthesiology, where the CL psychiatrist is employed full time since 2001. All patients, without exception, undergo psychological evaluation. In this way, it is possible to obtain an overview of the group of CRPS patients and relate them to the entire group of outpatients treated for pain.

This retrospective study describes the neuropathic and psychiatric aspects of CRPS patients and their treatment.

## 2. Patients and Methods

### 2.1. Patients

For this retrospective study, we collected data from CRPS patients examined at the Department of Anaesthesiology (Medical Faculty of Otto-von-Guericke University) between January 1, 2001, and June 30, 2014. Data were analysed after consultation with the Ethics Committee of Otto-von-Guericke University and in strict compliance with data protection by pseudonymization. The correctness of the referral diagnosis of CRPS for outpatient treatment was checked and confirmed at our clinic according to the clinical data and criteria published by Stanton-Hicks et al. [1], Jänig and Stanton-Hicks [7] and, after adoption by the IASP in 2010, according to the Budapest criteria [8]. A comparison of the criteria before and after 2010 is displayed in Table 1.

One of the central issues of the patients' medical history is pain quality and pain severity. The Visual Analogue Scale (VAS) is a 10 cm scale that measures the severity of pain. The scale has different descriptors at each end on a horizontal line (VAS 0 is no pain, and 10 is the worst pain). In addition to the current pain level (current VAS), the patients are interviewed for their maximum (maximum VAS), minimum (minimum VAS), and target VAS (“Which pain level would you accept if we say that we cannot reach VAS 0?”).

To complete the medical history, the CL psychiatrist questioned the patients in a semistructured interview according to AMDP to get an overview of their psychic state. To evaluate and classify the findings, a comparison was made with the entire group of pain outpatients with corresponding somatic and psychiatric diagnoses for the period 2001–June 2014.

### 2.2. Assessment and Questionnaires

#### 2.2.1. AMDP (Working Group for Methods and Documentation in Psychiatry)

The AMDP System is a Manual for the Assessment and Documentation of Psychopathology. The AMDP is an addition to a small number of instruments that have been created and tested in an international setting [10].

#### 2.2.2. SCL-90-R

The SCL-90-R assesses psychological distress in terms of nine primary symptom dimensions and three summary scores termed global scores. SCL-90-R test was applied to all patients involved in this study. Somatization (SOM), Obsessive-Compulsive (O-C), Anxiety (ANX), Hostility (HOS), Phobic Anxiety (PHOB), Paranoid Ideation (PAR), and Psychoticism (PSY) subscales of SCL-90-R test were performed, and general assessment of SCL-90-R test was performed by the means of Global Severity Index (GSI). Its score is found by the average of the ratings of all items (range 0–4). GSI results indicate general assessment of SCL-90-R questionnaire.

### 2.3. Statistical Analysis

Statistical analysis was conducted with IBM SPSS version 24 (Armonk, NY, USA).

At first, descriptive analyses were carried out. The associations between qualitative variables were investigated by contingency table analyses using Pearson's chi-square test. The Mann–Whitney test was applied for comparison between quantitative variables (VAS). Wilcoxon's paired test was used to ascertain differences in quantitative data (VAS) between the time of the last consultation and the time of admission. This was done separately for the different groups to be investigated (CRPS I/CRPS II, F-diagnosis yes/no).

In all statistical tests, an error probability of *α* of 0.05 was assumed (all *p* values < 0.05 are considered significant).

## 3. Results

### 3.1. Demographic Data

Since the consequent registration of patients with somatic and psychiatric diagnoses at the Department of Anaesthesiology of Magdeburg Medical Faculty began in 2001, 64 CRPS patients (18 males and 46 females) have been treated (effective date: June 30, 2014). From the time of admission onwards, patients' age ranged from 19 to 89 years, with a mean (±standard deviation) of 49.6 ± 13.1 years. It must be pointed out that 47 patients developed CRPS after surgery. The demographic data are presented in Table 2.

A questionnaire was used to obtain an overview of all treatments applied for each patient to date and current medication was noted. These were as follows:

1. Medical treatment (*n*=59)
2. Physical therapy with remedial gymnastics (*n*=48), lymphatic drainage (*n*=12), occupational therapy (*n*=8), manual treatment (*n*=7), electrotherapy (*n*=8), and transcutaneous electrical nerve stimulation (*n*=13)
3. Psychotherapy (*n*=8) and autogenic training (*n*=6)
4. Invasive treatments with intra-articular injection of corticosteroids (*n*=14), local anaesthesia (*n*=3), stellate ganglion block (*n*=2), spinal cord stimulation (*n*=2), and intravenous therapy with NMDA receptor antagonist (*n*=1)
5. Alternative medicine with acupuncture (*n*=7)

An overview of administered substance classes after optimization and stepwise adaptation of the therapy is presented in Table 3. The staff of our department substantially increased the administration of low-potency opioids and psychotropic drugs such as anticonvulsants and antidepressants.

Clinical examination and electrophysiological studies performed in all the patients revealed CRPS II in 21 patients and CRPS I in 43 patients. Neuropathic complaints (burning, stabbing, cutting, tingling, shooting, and electrifying sensations) were mentioned by 60 of the 64 patients.

### 3.2. SCL-90-R

Altogether, the GSI data of 47 patients (73.4%) were compared. For 17 patients, no data were available. GSI had a mean value of 53.8 ± 8.4 (median 55.0, range from 33 to 70). Thirty-seven patients (78.3%) had GSI scores ≤ 60 (no increased mental stress), whereas 10 patients (21.3%) had GSI scores > 60. These patients showed increased values (total values > 61) for anxiety (scale 5: 65.0 ±3.6) and depression (scale 4: 70.0 ± 4.6).

### 3.3. CRPS Patients with Psychiatric Diagnosis

Twenty-eight of 64 CRPS patients received a psychiatric diagnosis. For 36 patients (56.3%), the psychiatric findings were unremarkable. Seven of 28 patients with psychiatric diagnosis received a double diagnosis (25.0%), one patient a triple diagnosis (3.6%), and one patient a quadruple diagnosis (3.6%). The diagnoses are presented in Table 4.

### 3.3.1. Psychiatric Diagnoses—A Comparison between CRPS Patients and the Common Patient Cohort of the Department of Anaesthesiology

The psychiatric examinations of all the patients treated at the Department of Anaesthesiology (*n*=2439) gave the following picture:

1. The psychological findings of 805 patients (33%) were unremarkable.
2. 1634 patients (67%) had at least one psychiatric diagnosis.
3. 866 (35.5%) of the patients had two, 173 (7.1%) had three, 10 (0.4%) had four, and two patients (0.1%) had five psychiatric diagnoses at the same time.
4. Depression (F3) was diagnosed in 15.5% (*n*=378).
5. 207 (8.5%) of the patients abused pain killers or other substances.
6. 636 patients (26.1%) were affected by a neurotic spectrum disorder (F4 = conversion disorder, anxiety disorder, somatoform disorder, adjustment disorder, and hypochondriasis).

The CRPS patients and all the other pain outpatients of our department were compared for the frequency of psychiatric diagnoses. This comparison revealed that CRPS patients were less frequently affected by organic defects including symptomatic psychiatric disorders (F0), as well as by neurotic stress and somatoform disorders. No anxiety disorders were noted. They also more frequently suffered from personality and behavioral disorders (F6) without any psychopathological findings (*p* < 0.001). These differences especially apply to female patients (*p*=0.005).

Psychiatric findings classified as F3 and F4 (mood and adjustment disorders) of patients with somatic pain diagnoses (different somatic pain groups according to Klinger et al. [11]) in our pain clinic are presented in Table 5.

CRPS patients had the lowest proportion of psychiatric disorders and come after the group of patients with tumour pain. However, as far as the F3 depressions are concerned, their proportion is higher than the mean of the entire group of pain patients.

### 3.4. Reduction in Pain Severity

#### 3.4.1. Pain Severity in CRPS I and CRPS II Patients

**Figure 1:**
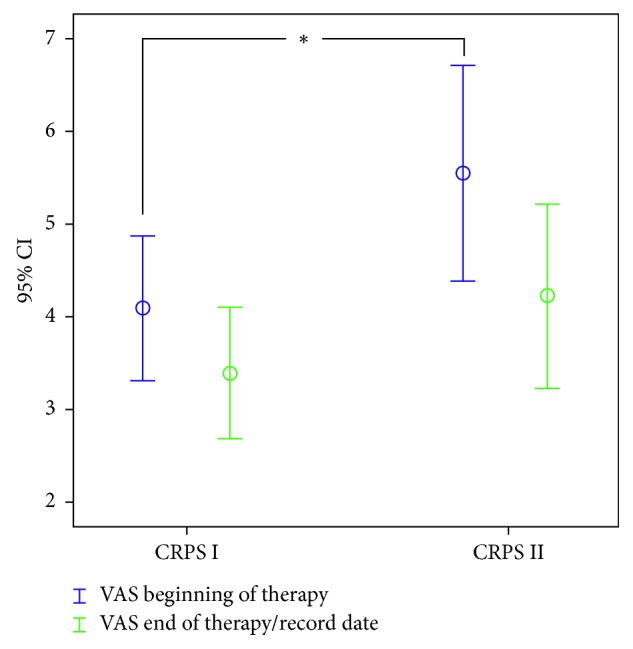
The course of average VAS between CRPS types I and II. Time point 1: beginning of therapy; time point 2: end of medication switch/record date; ^*∗*^significant difference between CRPS type I and type II at the beginning of therapy.

On admission, CRPS patients with nerve lesions had a significantly higher VAS level than those without (VAS 5.6 ± 2.5 versus VAS 4.1 ± 2.5, *p*=0.037). At the end of the medication switch, CRPS patients with nerve lesions reached a VAS pain level of 4.25 ± 2.2 and those without nerve lesions reached a VAS level of 3.39 ± 2.2 (Figure 1).

#### 3.4.2. Pain Severity in CRPS Patients with Psychiatric Diagnosis

There were significant differences in patients with psychiatric diagnosis on admission. The VAS data at presentation were as follows (psychiatric diagnosis yes/no):

  VAS current: 5.63/3.97 (*p*=0.031)
  VAS minimum: 4.0/2.63 (*p*=0.015)
  VAS target: 3.08/2.03 (*p*=0.008)

Here, the CRPS patients with psychiatric diagnosis showed significantly higher VAS values. No differences were found for VAS maximum (8.07/7.89; *p*=0.658) and VAS end (4.08/3.39; *p*=0.228).

**Figure 2:**
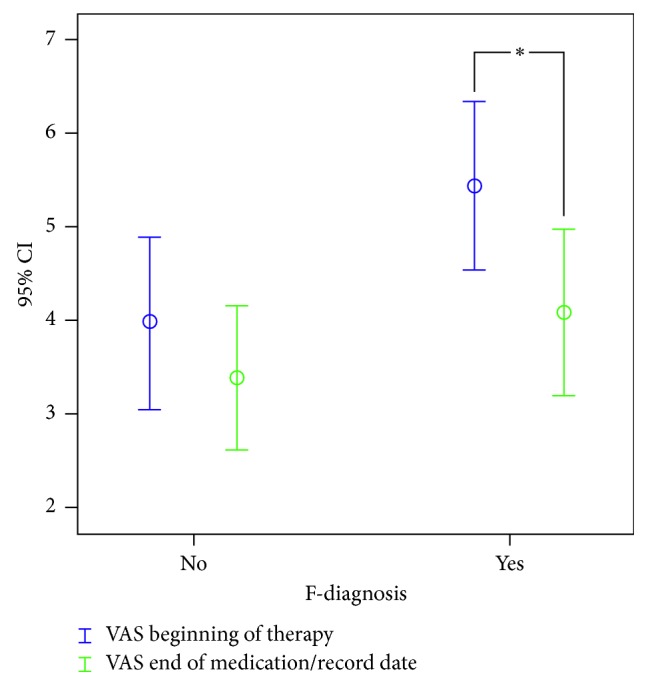
The course of average VAS between CRPS patient with or without F-diagnosis. Time point 1: beginning of therapy; time point 2: end of medication switch/record date; ^*∗*^significant difference to beginning of therapy.

Furthermore, patients with a psychiatric diagnosis showed significantly decreased VAS values at the end of therapy/record date (*p*=0.02) when compared to the baseline values (Figure 2).

## 4. Discussion

Consensus has been reached in the literature that CRPS is a serious disease characterized by a heterogeneous expression and a course that is difficult to predict. The reason for this lies in numerous influences, including psychological factors [12]. Therefore, it is necessary to search for factors that either favour the development of or are caused by CRPS, thus complicating the course. Therefore, early diagnosis is the best prerequisite for a good prognosis [13].

Although this retrospective study focused on psychological aspects, a multimodal approach to pain therapy was adhered to in all patients. This included physical therapy and lymphatic drainage when indicated.

This study did not concentrate on remarkable life events or coping strategies [12, 14], but rather placed emphasis on psychological factors and psychiatric diagnoses.

The majority of authors assume that in CRPS patients, psychological disorders and changes in the patients' behavior are to a greater extent a consequence of the pain rather than the cause [15]. Other study groups report that psychiatric disorders had already been diagnosed before CRPS occurs [16, 17].

Numerous case reports characterize CRPS patients as anxious, strained, irritated, emotionally labile, or depressive [18–20]. The description of these patients focused on either personal traits or psychological factors, such as depression or anxiety [21–24], which are presumed to occur more frequently in CRPS patients. This corresponds to the personal traits mentioned above. Anxiety, in particular, is associated with higher risk of developing CRPS after fractures [25]. According to Rommel et al. [3], increased anxiety is a factor that increases pain [25]. In our study though, the questionnaire SCL-90-R revealed that only a small patient cohort was affected by anxiety.

Psychiatric disorders, such as depressive mood disorders or anxiety disorders, have only rarely been investigated. Two study groups have tried to ascertain the proportion of psychiatric disorders in CRPS by using SCID for DSM-III and DSM-IV (Structured Clinical Interview for Diagnostic and Statistical Manual of Mental Disorders). In 1998, Monti et al. [13] compared pain diseases with a neuropathic component. They compared 25 patients with CRPS I and 25 patients with chronic back pain following a herniated disk using a structured clinical interview according to SCID for DSM-III. 20% and 24% of the patient groups fulfilled the criteria for a major depression (i.e., F3 depression). Of the nondepressed patients, 60% and 64% showed no sign of a personality disorder. Thus, no significant differences were noted.

In their most recent systematically performed psychiatric investigation performed in 2001, Rommel et al. [3] concentrated on the recognition of psychiatric disorders in CRPS. That study included 40 patients interviewed according to SCID for DSM-IV. The interview for that study was performed by an experienced psychologist. Thirty-seven of 40 patients were evaluated. Eight patients (22%) were found to be psychologically normal in the preliminary finding and at the time of examination. Rommel et al. registered adjustment disorders in twelve of the 37 patients and major depressions (F32.1) in five patients and summarized these deficiencies as psychiatric disorders occurring after CRPS. Due to the fact that depression episodes occurred in six patients before CRPS, in five patients after CRPS, and in seven patients in the further course, it has to be presumed that cyclothymic courses with interim inconspicuous stages (6/37 = 16.2%) were responsible. Furthermore, these patients constituted a minority compared to those affected by adjustment disorders (6 versus 12). The majority of patients showed signs of anxiety before onset of CRPS.

In our study, we registered a depression rate of 15.6%, which is similar to the percentage reported by Rommel et al. When compared to the entire group, anxiety disorders were not diagnosed.

In our department, both an anaesthesiologist and a permanently present CL psychiatrist take care of all the pain patients. This means that psychological findings are available for all patients (100%). Compared to the entire patient cohort of the Department of Anaesthesiology, the proportion of psychiatric disorders in CRPS patients is lower. A specific comparison with back pain and muscle-joint pain revealed that the proportion was considerably lower.

According to Feliu and Edwards [26], specific predisposing factors of the psychological or psychiatric kind which trigger or intensify CRPS do not exist for CRPS patients, nor can these factors be found in other patients with chronic pain.

However, compared to the common population, the CRPS patients of our study show

1. a higher prevalence (15.6%) for depression. The TACOS study in Germany (Transitions in Alcohol Consumption and Smoking) interviewed 4075 persons by telephone and found that 11.5% of the questioned participants were affected by major depression [27],
2. a higher prevalence for depression compared to the majority of somatic patients of the entire group,
3. that concerning depression, our data are similar to those of Rommel et al. [3].

Rommel et al. concluded that depressive mood disorders constitute a factor that promotes disease and intensifies pain. In our study, pain was more intense in patients with psychiatric disorders. Other studies also confirm that psychiatric disorders intensify pain in CRPS patients [28].

In general, patients with neuropathic pain have a lower incidence of depression than patients with other forms of physical pain. In our study population, 7.2% had a F3 diagnosis, while 15.9% of CRPS patients showed a F3 diagnosis (Table 5). Smith et al. found lower suicidal tendencies in patients with neuropathic pain compared to stomach pain and explain this by the concept that neuropathic pain has a physical and anatomic correlate [29].

In a publication from our study group, we could show that neuropathic pain has an incidence of 8.2% for F3 depression as opposed to lower back pain (14.0%) and muscle-joint pain (15.0%) in a population of 485 outpatients assessed over a consecutive 6-month period [30].

Maier et al. found in a general population a prevalence of 4.5% for F3 depression [31], whereas Elisei et al. located them at 17% [32].

Different therapies have been recommended for the treatment of CRPS. A large number of studies have investigated the analgesic effects of different therapy forms [33]. However, success can be achieved only by combination of different therapies and early diagnosis [34]. Another aspect needs to be added: Our study is the first to emphasize the bipolar efficacy spectrum of analgetically effective psychiatric drugs. As CRPS patients have comorbidity with F3 depression, these patients should be treated with dual active psychotropic drugs, that is, serotonin-noradrenaline reuptake inhibitors (SNRIs) as opposed to selective serotonin reuptake inhibitors (SSRIs) [30]. In the treatment of depression and pain, SNRIs have a direct analgetic influence on pain and the pain is reduced indirectly by increasing the threshold for neuralgia. Therefore, the treatment with dual active psychotropic drugs could have facilitated the reduction in VAS pain scores we found.

Due to the association between depression and suicidal tendencies among pain patients [35–37], surveillance by nonpsychiatrists [38, 39] and increased psychological care [40] are recommended in current literature.

### 4.1. Limitations of the Study

This study only compared the psychiatric diagnoses of CRPS patients with those of other chronic pain patients in an outpatient clinic of a university hospital. It was not the aim of this study to perform an expansive psychological assessment or to systematically determine radical and stressing life events and their timely assignment to the disease process.

Also, the diagnostic criteria of CRPS were revised and adopted by the IASP in 2010. The Budapest Criteria have a sensitivity nearly identical to that for the IASP criteria, but with substantially improved specificity, and furthermore lead to improved diagnostic consistency between clinicians [8].

## 5. Conclusions

CRPS is a syndrome that offers an extremely variable picture. Emphasis is placed on two psychiatric factors (anxiety and depression). Some authors assign a repeatedly noted anxiety to a type of personality, whereas others regard this as a clue to anxiety disorders and their differential diagnoses [41]. In our retrospective study, increased anxiety was noted only in a small number of patients.

The depressions were assigned to affective disorders (F3) in two publications based on SCID for DSM-III [13] and, respectively, on SCID for DSM-IV [3]. Chronic pain patients in general and CRPS patients in particular are affected by a larger number of depressions compared to the normal population.

However, as the number of cases is very small, this conclusion should be regarded with caution. But those data are confirmed by current investigations and by comparison with all disease courses evaluated by the Department of Anaesthesiology to date. As depressions may lead to a higher rate of suicidality in CRPS patients [35, 37], psychological and psychiatric treatment is recommended as early as possible.

## Figures and Tables

**Table 1:** IASP criteria for the diagnosis of CRPS before and after 2010.

IASP diagnostic criteria for complex regional pain syndrome (CRPS)^*∗*^ up to 2010 [9]	IASP diagnostic criteria for complex regional pain syndrome (CRPS) after 2010 [8]
(1) The presence of an initiating noxious event, or a cause of immobilization^†^	(1) Continuing pain, which is disproportionate to any inciting event

(2) Continuing pain, allodynia, or hyperalgesia in which the pain is disproportionate to any known inciting event	(2) Must report at least one symptom in three of the four following categories:
	Sensory: reports of hyperalgesia and/or allodynia
	Vasomotor: reports of temperature asymmetry and/or skin color changes and/or skin color asymmetry. Sudomotor/edema: reports of edema and/or sweating changes and/or sweating asymmetry. Motor/trophic: reports of decreased range of motion and/or motor dysfunction (weakness, tremor, dystonia) and/or trophic changes (hair, nails, skin)

(3) Evidence at some time of edema, changes in skin blood flow, or abnormal sudomotor activity in the region of pain (can be sign or symptom)	(3) Must display at least one sign^#^ at the time of evaluation in two or more of the following categories: Sensory: evidence of hyperalgesia (to pinprick) and/or allodynia (to light touch and/or deep somatic pressure and/or joint movement)
	Vasomotor: evidence of temperature asymmetry and/or skin color changes and/or asymmetry. Sudomotor/edema: evidence of edema and/or sweating changes and/or sweating asymmetry. Motor/trophic: evidence of decreased range of motion and/or motor dysfunction (weakness, tremor, dystonia) and/or trophic changes (hair, nails, skin)

(4) This diagnosis is excluded by the existence of other conditions that would otherwise account for the degree of pain and dysfunction	(4) There is no other diagnosis that better explains the signs and symptoms

^∗^If seen without “major nerve damage,” diagnose CRPS I; if seen in the presence of “major nerve damage,” diagnose CRPS II. ^#^A sign is counted only if it is observed at the time of diagnosis. ^†^Not required for diagnosis; 5–10% of patients will not have this.

**Table 2:** Demographic data with mean value (±SD); median (range).

Male/female	18/46
Age at onset of pain	49.6 years (±13.1 years)
Age at last consultation	53.6 years (±12.8 years)
Time period between onset of pain and outpatient admission	1.6 years (±2.5); 0.5 years (immediately,13.3 years)
Time period between diagnosis and outpatient presentation	0.4 years (±1.0); 0.02 years (immediately, 7.5 years)
Treatment period	2.4 years (±3.6); 0.6 years (one consultation up to 15 years)
Number of consultations	23 (±37); 9 (1,190 consultations)
Upper/lower extremities	56/8
CRPS type I/type II	43/21
Number of patients with surgery before onset of CRPS	47

The VAS value of all 64 patients was 5.0 ± 2.5. Value at the end of therapy: 3.6 ± 2.1 (*p*=0.226).

**Table 3:** Overview of drug therapy distribution of drugs before outpatient admission and after adaptation of medication.

Drugs	Before admission (*n*)	At present or at end of therapy (*n*)
Low-potency opioids	24	40
High-potency opioids	6	11
NSAID	45	5
Anticonvulsant drugs	10	30^*∗*^
Antidepressant drugs	6	39^*∗∗*^
Neuroleptics	0	2
Glucocorticoids	4	0
Calcitonin	3	1

^*∗*^Gabapentin, *n*=15; pregabalin, *n*=9; ^*∗∗*^amitriptyline, *n*=20; mirtazapine, *n*=7; venlafaxine, *n*=6.

**Table 4:** Psychiatric diagnoses of ICD-10 for CRPS patients.

Diagnostic group	Commentary	Number of patients
*F0*	*Organic, including symptomatic, mental disorders*	*Total 3*
F07	Organic personality disorder	3

*F1*	*Mental and behavioral disorders due to psychoactive substance use*	*Total 6*
F10	Mental and behavioral disorders due to use of alcohol	3
F13.1	Harmful use of sedatives or hypnotics	1
F17.2	Dependence syndrome due to use of tobacco	2

*F2*	*Schizophrenia, schizotypal, and delusional disorders*	*Total 2*
F24	Induced delusional disorder	1
F25.2	Schizoaffective disorder, mixed type	1

*F3*	*Mood (affective) disorders*	*Total 10*
F32.0	Mild depressive episode	1
F32.1	Moderate depressive episode	3
F34.0	Cyclothymia	2
F34.1	Dysthymia	4

*F4*	*Neurotic, stress-related, and somatoform disorders*	*Total 11*
F42.1	Predominantly compulsive acts (obsessional rituals)	1
F43.2	Adjustment disorders	6
F44	Dissociative (conversion) disorders	2
F45.0	Somatization disorder	1
F45.4	Persistent somatoform pain disorder	1

*F6*	*Disorders of adult personality and behavior*	*Total 7*
F60.4	Histrionic personality disorder	1
F60.7	Dependent personality disorder	1
F60.8	Other specific personality disorders	2
F60.9	Personality disorder, unspecified	1
F62.8	Chronic pain personality syndrome	1
F66.0	Sexual maturation disorder	1

*F9*	*Behavioral and emotional disorders with onset usually occurring in childhood and adolescence*	*Total 1*

F95.1	Chronic motor or vocal tic disorder	1

**Table 5:** Percentage of mood disorders (F3) and neurotic spectrum disorders (F4) in different somatic pain groups according to Klinger, arranged in ascending order for F3.

Diagnosis	*n*	F3 (%)	F4 (%)	Total percentage of F-diagnoses (%)
Vascular pain in limbs	18	5.6	16.7	61.1
Neuropathia totally	276	7.2	15.9	55.8
Cancer pain	148	9.5	7.4	39.2
Chest pain	43	11.6	34.9	62.8
Muscle-joint pain	456	13.4	27.2	65.6
Chronic low back pain with radiculopathia	87	14.9	19.5	64.4
*CRPS*	*64*	*15.6*	*17.2*	*43.8*
Abdominal pain	88	15.9	36.4	81.8
Chronic low back pain without radiculopathia	905	16.9	31.4	71.7
Headache and facial pain	185	20.0	22.7	62.2
Fibromyalgia and panalgesia	171	25.7	31.6	86.5
Cenesthesia	62	25.8	16.1	72.6

## Data Availability

The data used to support the findings of this study are available from the corresponding author upon request.
